# A novel hybrid segmentation method coupled with deep learning for coronary artery extraction from coronary CT angiography

**DOI:** 10.1007/s10554-026-03643-7

**Published:** 2026-02-26

**Authors:** Daebeom Park, Soon-Sung Kwon, Yoon A Kim, Joo Young Kim, Baren Jeong, Eun-Ah Park, Yoon Seong Lee, Whal Lee

**Affiliations:** 1 Department of Clinical Medical Sciences, Seoul National University College of Medicine, Seoul, Korea; 2 Department of Radiology, Seoul National University Hospital, 101 Daehak-ro, Jongno-gu, Seoul, 03080 Korea; 3 Institute of Convergence Medicine with Innovative Technology, Seoul, Korea; 4 AI Medic Inc., Seoul, Korea

**Keywords:** Coronary computed tomographic angiography (CCTA), Deep learning, Coronary artery segmentation, Coronary artery disease, Hybrid method

## Abstract

Coronary computed tomographic angiography (CCTA) is a non-invasive imaging technique widely used for diagnosing coronary artery disease (CAD), one of the leading causes of mortality in developed countries. Accurate and automatic segmentation of coronary arteries from CCTA is essential for extracting both anatomical and pathological information. Existing deep learning methods suffer from noise artifacts and vessel discontinuities, while classical image processing methods including fixed Hounsfield unit (HU) threshold are highly dependent on scanner characteristics. In this study, we proposed a novel hybrid method that integrated deep learning with our unique mathematical integration of image processing filters, featuring a contour detection algorithm that exploited intensity gradients. The performance of our method was quantitatively evaluated using CCTA scans from 84 patients (internal validation set) and 40 patients from a public dataset (external validation set), with segmentation results compared against manually annotated reference data. We also evaluated existing deep learning-only and classical fixed HU threshold methods against the same reference data for comparison. Our hybrid method demonstrated superior performance with a Dice score of 0.92 (95% confidence interval [CI]: 0.91–0.93), significantly outperforming deep learning-only (0.68, 95% CI: 0.66–0.69, p < 0.001) and fixed HU threshold methods (0.55, 95% CI: 0.53–0.56, p < 0.001). External validation on public datasets confirmed significantly better performance with a Dice score of 0.82 (95% CI: 0.81–0.82) compared to deep learning-only (0.76, 95% CI: 0.74–0.77, p < 0.001) and fixed HU threshold methods (0.76, 95% CI: 0.75–0.77, p < 0.001). These results indicate that our hybrid method enables robust and consistent automatic coronary artery segmentation from CCTA, demonstrating potential to aid CAD assessment in clinical practice.

**Supplementary Information:**

The online version contains supplementary material available at 10.1007/s10554-026-03643-7.

## Introduction

Coronary artery disease (CAD) is a leading cause of mortality worldwide, and its prevalence is steadily increasing [1–3]. Coronary computed tomographic angiography (CCTA) has been widely used as a non-invasive diagnostic tool for CAD, with its applicability further enhanced by advancements in 3-dimensional (3D) volume rendering technology [4]. A crucial step in using CCTA for CAD assessment is the segmentation of coronary arteries, which enables the reconstruction of their 3D geometry [5, 6].

Previous studies have proposed various approaches for coronary artery segmentation, which can be broadly categorized into two types: methods that achieve full automation through deep learning techniques [5, 7, 8] and methods based on classical image processing techniques for segmentation [9].

However, both approaches have several limitations. Deep learning-only methods require extensive annotated datasets, which are labor-intensive to acquire and subject to inter-operator variability [8, 10]. Additionally, these methods face domain-specific challenges including noisy outputs, calcification misclassification, and vessel connectivity issues that result in incomplete segmentation [11–13]. On the other hand, classical image processing methods typically rely on fixed Hounsfield unit (HU) thresholds, region growing, and graph cuts to differentiate coronary arteries from surrounding tissues [14–16]. However, in stenotic or distal regions, the HU intensity of surrounding tissues is often similar to that of coronary arteries, resulting in segmentation errors [17–19]. Consequently, many existing methods remain semi-automatic, requiring manual intervention to refine segmentation results, which increases both processing time and inter-operator variability [20].

To address these limitations, we proposed a novel hybrid method that combines deep learning-based initial segmentation with our unique mathematical integration of traditional image processing filters for fully automated coronary artery extraction. This hybrid method was designed to target noisy outputs and vessel connectivity issues, while our mathematical integration was developed to handle calcification misclassification and segmentation errors in distal branches and stenotic regions. The objective of this study was to validate a hybrid segmentation method for coronary artery extraction from CCTA images to observe its potential in aiding CAD assessment in clinical practice.

## Methods

### Ethics statement

The Institutional Review Board (IRB) of Seoul National University Hospital reviewed and approved the retrospective design of this study (IRB No. 2506-055-1647). The need to obtain informed consent was waived by the Institutional Review Board of Seoul National University Hospital. All experiments and methods were performed in accordance with relevant guidelines and regulations.

### Patient dataset

This retrospective study initially included 90 patients who underwent CCTA at Seoul National University Hospital. Six patients were excluded due to poor CT image quality, resulting in a final cohort of 84 patients for analysis. Demographic and clinical characteristics of the study population are summarized in Table 1. Detailed case-by-case information on stenosis severity and calcium scores is provided in Supplementary Table S1.

**Table 1:** Baseline demographic and clinical characteristics of study patients

Variables	Total population (n = 84)
Male (%)	52.4
Age (years)	65.2 ± 10.5
Height (cm)	162.3 ± 8.6
Weight (kg)	64.9 ± 13.2
Body mass index (kg/m^2^)	24.5 ± 3.8

Values are presented as mean ± standard deviation

For external validation, 40 additional CCTA cases were obtained from the ASOCA (Automatic Segmentation of Coronary Arteries) challenge public dataset (Coronary Atlas, https://www.coronaryatlas.org/) [21]. This cohort included 20 patients with coronary disease and 20 patients without disease as determined by cardiologist assessment.

### CCTA image acquisition and reconstruction

CCTA examinations were performed using either a third-generation dual-source CT scanner (SOMATOM Force; Siemens Healthineers, Forchheim, Germany; n = 40) or a wide-detector single-source CT scanner (Revolution Apex; GE Healthcare; n = 44). Tube voltage (70–120 kVp) and current were automatically optimized using kilovoltage peak selection software (CARE kV; Siemens Healthineers) and automatic exposure control (Care Dose 4D; Siemens Healthineers). Image reconstruction was performed with slice thickness of 0.625–0.75 mm, increments of 0.5–0.7 mm, and a medium soft convolution kernel utilizing iterative reconstruction algorithms. All CCTA datasets were acquired during cardiac phases with minimal motion, typically during end-systolic or mid-diastolic phases. Detailed imaging acquisition parameters and specifications are summarized in Supplementary Table S2.

For the external validation dataset, imaging was conducted using a 64-slice GE LightSpeed CT scanner employing retrospective electrocardiographic gating. Image reconstruction targeted the late diastolic phase, with pharmacological heart rate control using beta blockers to achieve rates below 60 beats per minute. Contrast enhancement was achieved using 60–80 ml of Omnipaque 350. The resulting images demonstrated anisotropic spatial resolution, featuring 0.3–0.4 mm in-plane pixel spacing and 0.625 mm slice thickness [21].

### A novel hybrid segmentation algorithm coupled with deep learning

**Fig. 1:**
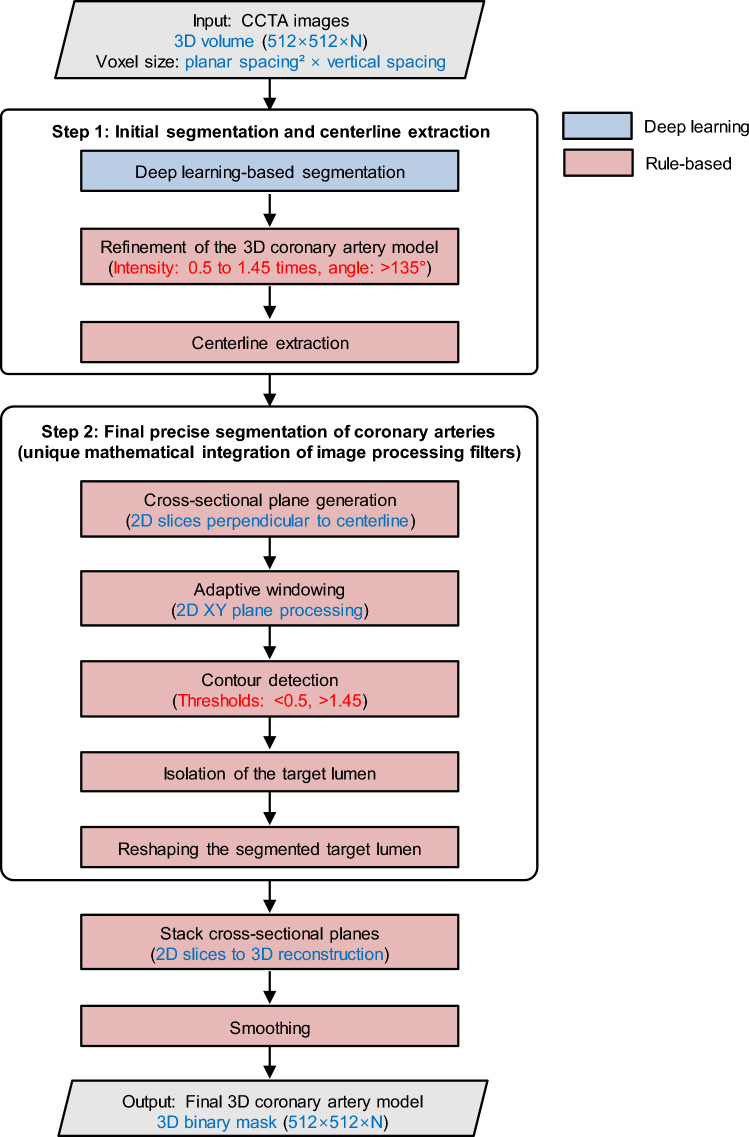
Flowchart of the hybrid coronary artery segmentation algorithm. The workflow is organized into two main steps using standard flowchart symbols: parallelograms represent input/output data, and rectangles represent processing operations. Step 1 performs initial segmentation and centerline extraction, beginning with deep learning-based segmentation (blue boxes) followed by rule-based refinement and centerline extraction (red boxes). Step 2 conducts final precise segmentation through unique mathematical integration of image processing filters (red boxes). The algorithm begins with 3-dimensional CCTA volume input, applies V-Net segmentation, refines using intensity (0.5–1.45 times component mean intensity) and angle (> 135°) thresholds, transitions to 2-dimensional cross-sectional analysis with contour detection using intensity thresholds (< 0.5 times, > 1.45 times seed intensity), and reconstructs into final 3-dimensional binary masks. Key numerical parameters are highlighted in red text, while data types and shapes are shown in blue text. 2D = 2-dimensional; 3D = 3-dimensional

The lumen segmentation was performed using a dedicated version of the software platform (HeartMedi + 1.0; AI Medic Inc., Seoul, South Korea). The overall workflow of our hybrid segmentation algorithm is illustrated in Fig. 1, which demonstrates a systematic two-step approach combining deep learning-based initial segmentation with rule-based refinement and our unique mathematical integration of image processing filters to produce final 3D coronary artery binary masks.

#### (i) Step 1: Initial segmentation and centerline extraction

**Fig. 2.**
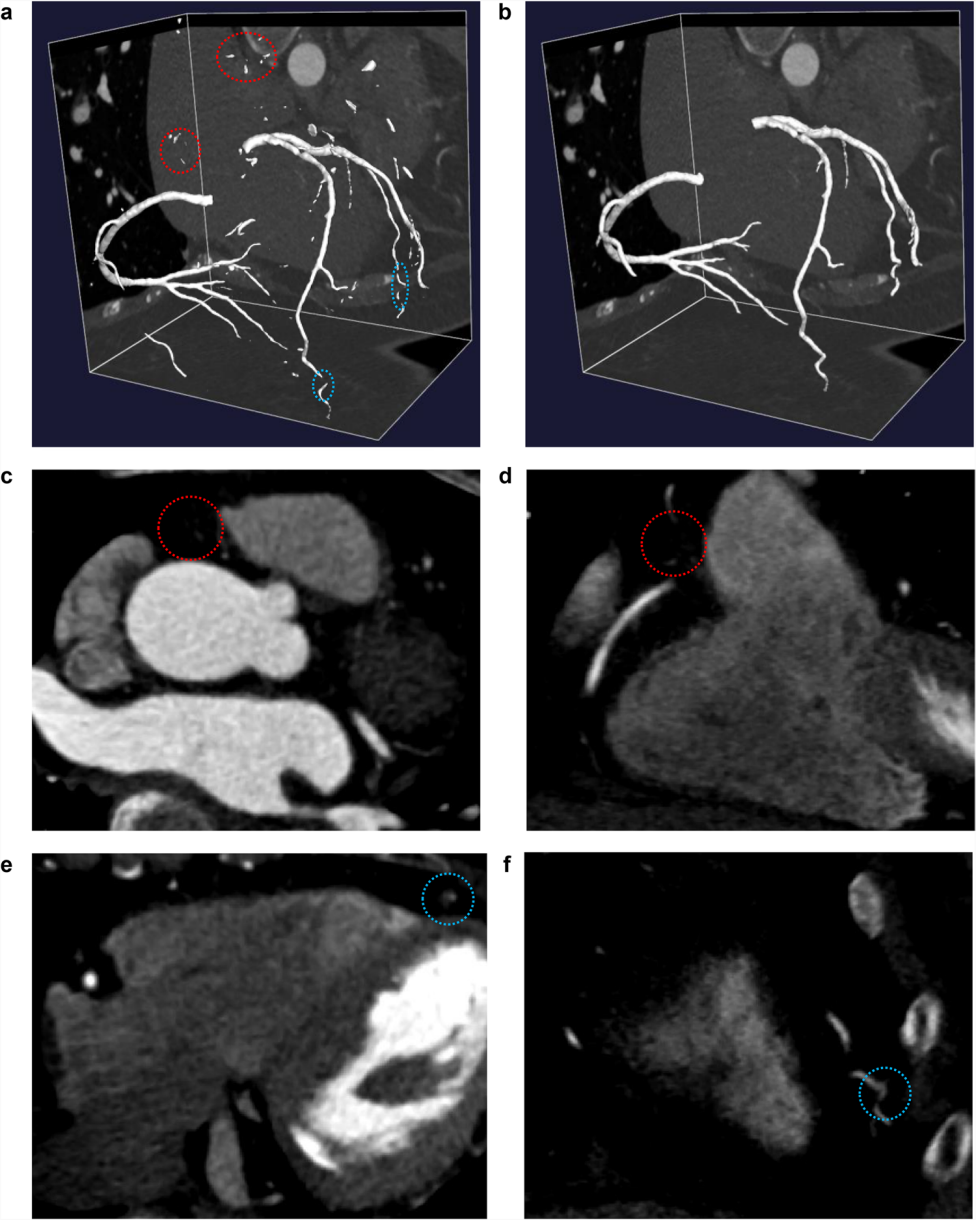
3-dimensional coronary artery segmentation outputs with corresponding 2-dimensional views. **a** Deep learning-generated segmentation output with noise (red dotted circles) and missing vessels (blue dotted circles). **b** Processed segmentation output after noise removal and vessel connection. **c**, **d** Representative axial (**c**) and coronal (**d**) views demonstrating noise artifacts (red dotted circles) that were removed during post-processing. **e**, **f** Representative axial (**e**) and coronal (**f**) views showing missing vessel segments (blue dotted circles) that were connected through our refinement algorithm

We utilized the V-Net, a well-established fully convolutional neural network specifically designed for segmenting volumetric medical images [22]. The V-Net was trained on 231,219 manually segmented coronary artery CT images from 457 patients across five institutions in South Korea (Supplementary Method S1). The current study cohort (n = 84) was completely independent from all training, validation, and internal testing sets, serving as an independent validation cohort for the trained model. The deep learning-generated segmentation served as an initial mask to guide the seed point placement and region-of-interest definition. Figure 2a illustrates the 3D coronary artery segmentation output generated using this deep learning technique.

The deep learning segmentation output consisted of multiple disconnected components rather than two complete coronary trees. To connect missing vessels and eliminate noise, component properties such as centerline distance, angle, directional consistency, and intensity characteristics were analyzed (Supplementary Method S2). Components were connected when specific geometric and intensity criteria were met [6, 23, 24], while venous structures were identified and removed based on anatomical trajectory patterns. Finally, two largest components identified as left coronary artery (LCA) and right coronary artery (RCA) were obtained, and remaining small disconnected components were subsequently removed as noise (Fig. 2b).

To further utilize the spatial and geometric information from the refined 3D coronary artery segmentation output and to achieve more accurate vessel boundary extraction, we extracted the centerline traversing through the coronary arteries (Supplementary Figure S1a). The centerline extraction method was based on the algorithm proposed by Antiga et al. [25] and was employed as seed points for the final precise segmentation step.

#### (ii) Step 2: Final precise segmentation of coronary arteries using our unique mathematical integration of image processing filters

All parameters and algorithmic procedures in this refinement step were predetermined and fixed prior to evaluation, with no optimization performed on the test datasets to ensure unbiased performance assessment.

**Fig. 3:**
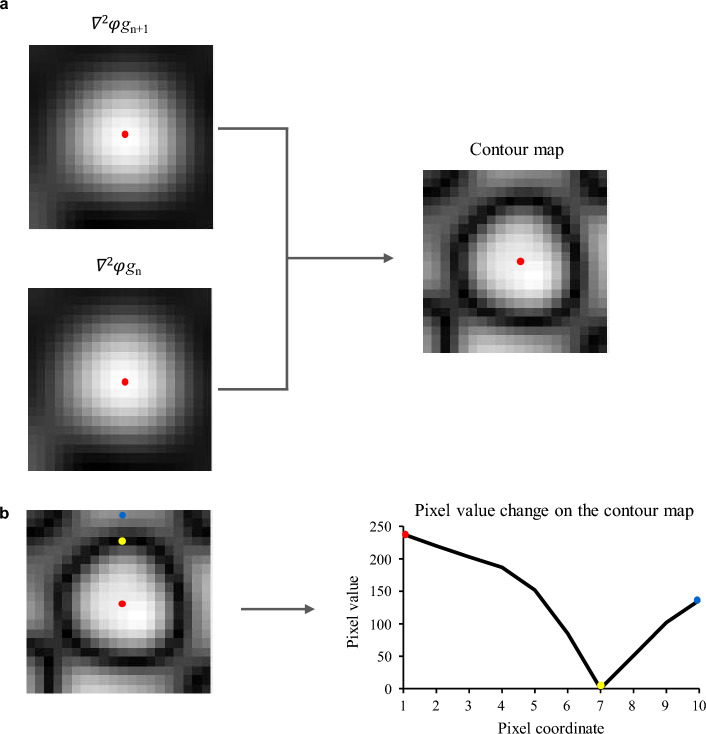
Edge detection using the contour map. **a** Generation of the contour map (see Methods for details). The red dot represents the seed point derived from the centerline information. **b** The graph showing pixel values along the path from the red to the blue dot on the contour map. The pixel value at the edge, represented by the yellow dot, is minimum compared to the values inside and outside of the target lumen

The final segmentation process began with the generation of cross-sectional planes along the centerline extracted in the first step (Supplementary Figure S1b). Each cross-sectional plane contained the target lumen at its center, and a central point within the lumen served as a seed for applying various image processing filters (Fig. 3c).

To facilitate coronary artery segmentation and prevent segmentation from extending to surrounding non-target structures, an adaptive window was applied within each cross-sectional plane to eliminate non-target regions. Following established theoretical principles for sequential filter application, Gaussian, weighted average (Supplementary Equation S1) [26–28], and Laplacian (Supplementary Equation S2) [27, 29] filters were systematically applied. This sequential application of filters isolated the target lumen from the background (Supplementary Figure S1d) to generate the final adaptive window (Supplementary Figure S1e). Detailed methodology for adaptive window generation is provided in Supplementary Method S3.

Once the adaptive window was set, a contour detection algorithm was applied to identify the boundaries of the target lumen. Since fixed thresholds were inadequate for precise boundary detection, we developed a novel approach exploiting the fact that vessel boundaries show maximum intensity gradients with minimal response to weighted average filters smoothing. A contour map was constructed, with pixel values calculated as follows (Fig. 3a):

where ${\nabla }^{2}$ denoted the Laplacian function, $\varphi$ represented the Gaussian function, and *n* indicated the number of weighted average filters applied, while individual components are established image processing techniques, this specific mathematical combination for coronary artery boundary detection represents our unique contribution. Figure 3b illustrates that the pixel values along the contour were lower than those inside and outside the target lumen. To detect the contour, the algorithm searched for inflection points in all directions from the seed point on the contour map.

To ensure accurate contour identification and avoid misclassification of intraluminal and calcified regions, the algorithm evaluated the original intensity values of the seed point. Inflection points with values significantly lower (< 0.5 times seed intensity) or higher (> 1.45 times seed intensity) than those of the seed point were selected as contour boundaries, based on established principles for distinguishing vessel boundaries from intraluminal [6] and calcified regions [24]. However, in cases where the seed point’s intensity was below a certain threshold due to luminal stenosis, false boundaries outside the lumen could be detected. In such cases, all inflection points were classified as lumen contours.

**Fig. 4:**
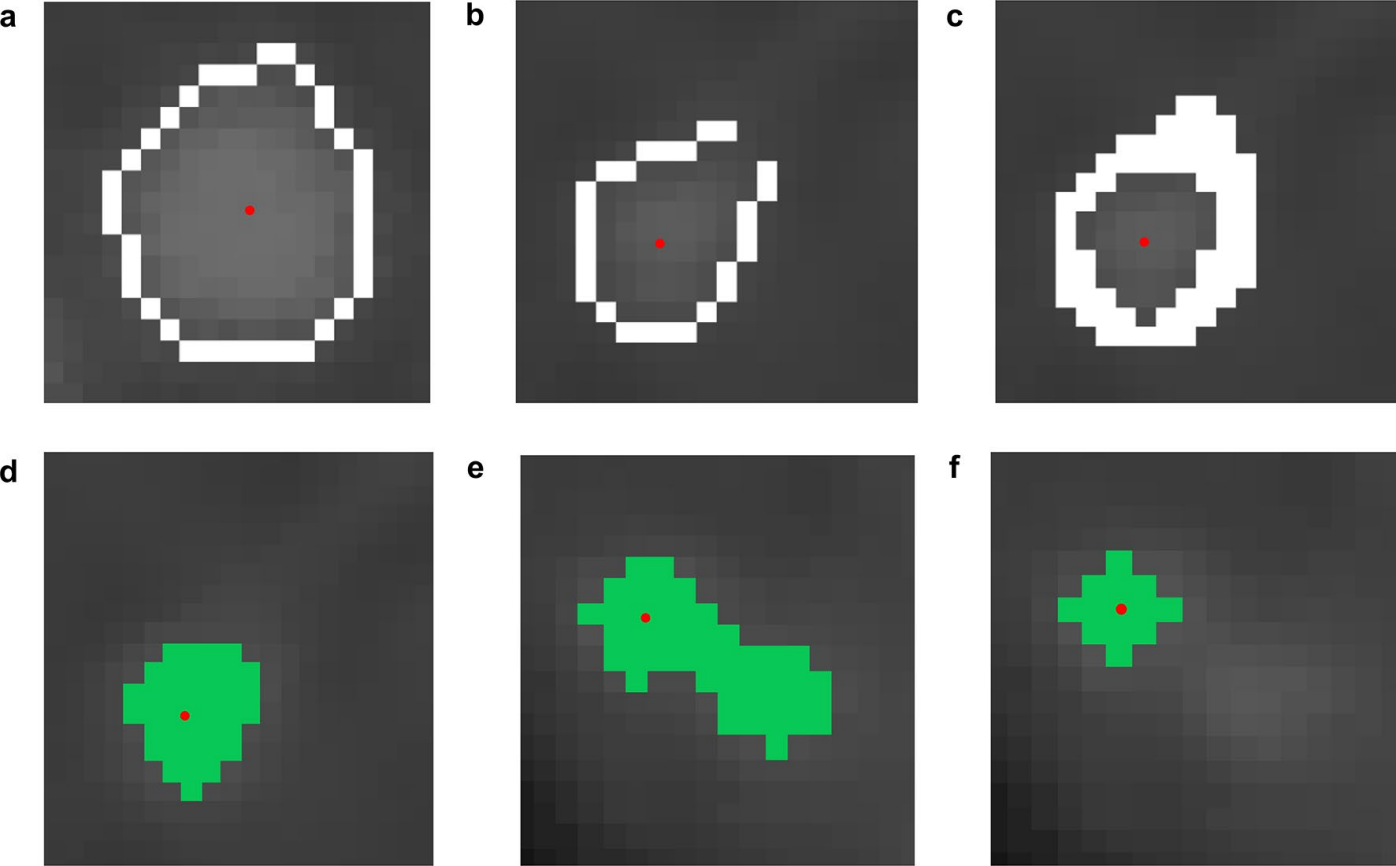
Representative examples of the method used in the final precise segmentation algorithm. **a** Successful contour detection result showing clear boundary identification of the target lumen (in white). **b** Challenging case where incomplete contour detection occurs due to surrounding structures with similar pixel intensities to the lumen boundary (in white). **c** Refined result from case **b** after applying upper and lower bounds of standard deviations from the average of the original pixel values along the contour (in white). **d** Segmentation result using the flood fill algorithm (in green). **e** Example case where the flood fill algorithm occasionally produced two independent lumens (in green). **f** Corrected result after applying the reshaping process to case (**e**) (in green). The red dot represents the seed point derived from the centerline information

The initial contour detection results are presented in Fig. 4a-b. However, incomplete lumen isolation may occur due to surrounding structures exhibiting similar pixel intensities to the lumen boundary (Fig. 4b). To address this, the algorithm refined the target lumen by separately processing low-intensity and high-intensity contour pixels. For each intensity group (low-intensity: < 0.5 times seed intensity; high-intensity: > 1.45 times seed intensity), the upper and lower bounds were calculated using the standard deviation of the respective contour pixel values (Supplementary Equation S3). Pixels with values within their respective bounds were further classified as lumen contours (Fig. 4c). Once the target lumen was fully isolated, segmentation was completed using the flood-fill algorithm, a widely used digital image processing technique (Fig. 4d) [30, 31].

The flood-fill segmentation process occasionally produced two independent lumens (Fig. 4e). Additionally, the cross-sectional shape of the coronary artery lumen is typically circular [32], but the segmentation output could include non-target tissues, resulting in an irregular lumen shape. To correct this, the algorithm calculated the radius of the segmented lumen in all directions from the seed point. If any radius was abnormally large or small, it was replaced with the mean of the remaining radii (Fig. 4f). This process ensured a circular lumen shape with uniform radii.

**Fig. 5:**
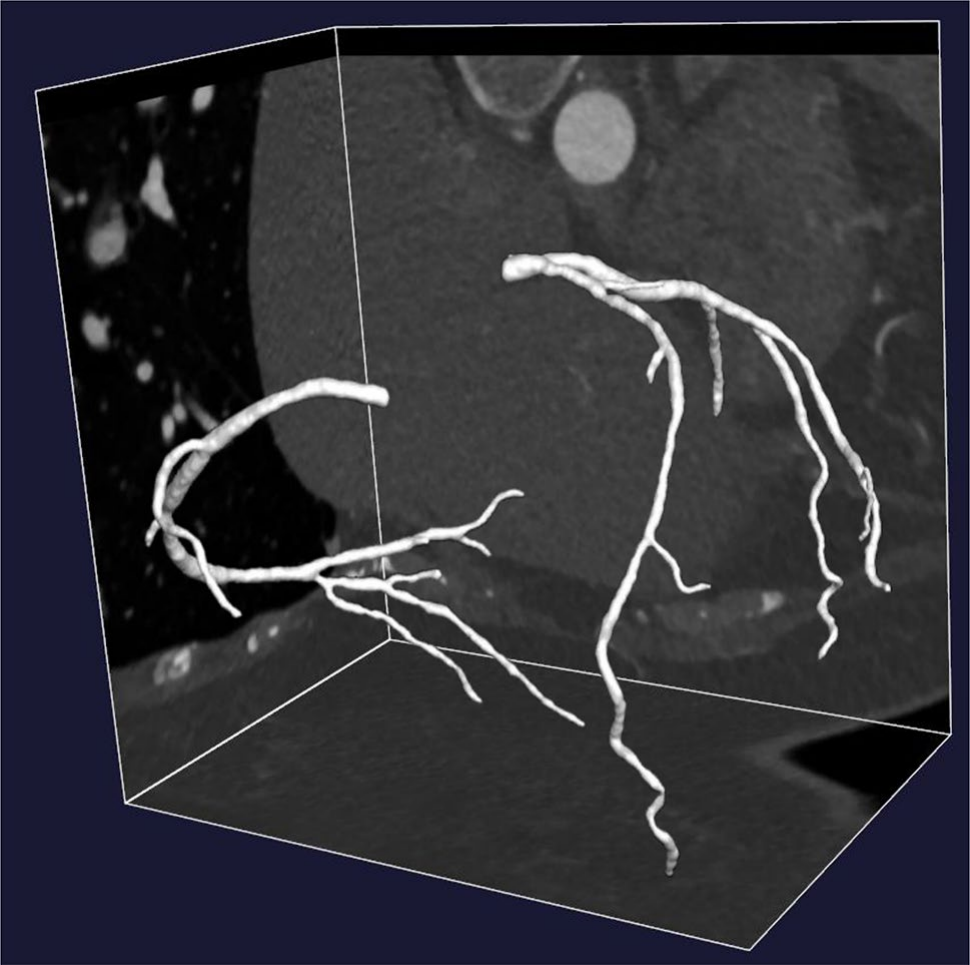
A reconstructed coronary artery segmentation output generated by the final proposed segmentation method

The reshaped lumens from all cross-sectional planes were stacked to reconstruct the 3D coronary artery segmentation output, followed by morphological smoothing [30, 33, 34] to eliminate surface discontinuities (Fig. 5, Supplementary Method S4).

### Performance evaluation

To evaluate the performance of the proposed segmentation method, a quantitative comparison was conducted against manually segmented reference data at the pixel level. Manual segmentation was performed by an experienced radiologic technologist with over 5 years of clinical experience using Rapidia 3D version 2.8 (INFINITT Co., Ltd.). All annotations were double-checked for quality and consistency.

To assess the contribution of our hybrid method, we performed ablation analysis comparing deep learning-only (V-Net alone in Step 1) and the proposed hybrid method (Step 2 with Step 1). Additionally, we compared our method against the fixed HU threshold method (fixed HU threshold with V-Net in Step 1), which applied HU threshold of 120 or above for lumen boundary detection [9].

Pixel-level segmentation performance was assessed using recall, precision, Dice score, and 95th percentile Hausdorff Distance (HD95). Recall measured the sensitivity of the segmentation by calculating the proportion of correctly identified positive pixels, while precision evaluated the positive predictive value by determining the proportion of predicted positive pixels that are actually correct. The Dice score quantified the overall overlap between predicted and ground truth segmentations (Supplementary Equation S4) [35], and HD95 measured boundary accuracy between segmented and reference contours [21].

Additional vessel structure-specific metrics were evaluated to assess the clinical relevance of the segmentation results. These included branch detection rate to quantify correctly identified vessel branches, vessel continuity to evaluate preservation of vessel connectivity, centerline distance (mean and maximum) to measure spatial accuracy of vessel pathways, and centerline overlap calculated using HD95 to measure the accuracy of extracted vessel centerlines.

### Subgroup analysis

To evaluate segmentation performance across different clinical and technical parameters, patients were categorized by stenosis degree (< 30% stenosis, n = 47 vs ≥ 30% stenosis, n = 37), coronary calcium score (calcium score ≤ 100, n = 43 vs calcium score > 100, n = 41), and CT scanner type (SOMATOM Force, n = 39 vs Revolution Apex, n = 45) (Supplementary Table S1). Segmentation performance metrics were calculated for each subgroup.

### Statistical analysis

Paired t-tests were performed to evaluate differences between the proposed hybrid method and deep learning-only method, as well as between the proposed method and classical fixed HU threshold method, using the same patient cohorts. For subgroup analysis, differences in segmentation performance between subgroups were assessed using independent t-test. A p-value < 0.05 was considered statistically significant. All analyses were performed using R version 4.2.0.

## Results

### Diagnostic performance analysis (internal validation)

To evaluate the performance of the proposed hybrid method, a quantitative comparison was conducted against manually annotated reference data. Supplementary Figure S2 demonstrated representative cases showing original scan, ground-truth segmentation, and proposed hybrid method results for direct comparison.

Our hybrid method achieved superior performance with Dice score of 0.92 (95% CI: 0.91–0.93) compared to deep learning-only (0.68, p < 0.001) and fixed HU threshold methods (0.55, p < 0.001). The method demonstrated significant improvements in vessel continuity (1.00 vs 0.07 for deep learning-only, p < 0.001) and precision (0.93 vs 0.39 for fixed HU threshold, p < 0.001). Complete comparative results including vessel structure-specific metrics such as branch detection rate, vessel continuity, and centerline overlap are presented in Table 2, with mean and maximum centerline distance information provided in Supplementary Table S3.

**Table 2:** Quantitative performance comparison of coronary artery segmentation methods on internal validation set

Metric	Proposed hybrid method	Deep learning-only method	p value	Fixed HU threshold method	p value
Dice score	0.92 (0.91–0.93)	0.68 (0.66–0.69)	< 0.001	0.55 (0.53–0.56)	< 0.001
Recall	0.91 (0.90–0.92)	0.90 (0.89–0.91)	0.047	0.91 (0.89–0.92)	0.490
Precision	0.93 (0.92–0.94)	0.55 (0.53–0.57)	< 0.001	0.39 (0.38–0.41)	< 0.001
HD95 (mm)	7.61 (5.22–10.00)	8.85 (7.40–10.30)	0.281	14.83 (12.41–17.26)	< 0.001
Branch detection rate	0.81 (0.78–0.84)	1.22 (1.16–1.29)	< 0.001	0.94 (0.88–1.00)	< 0.001
Vessel continuity	1.00 (1.00–1.00)	0.07 (− 0.12–0.25)	< 0.001	1.00 (1.00–1.00)	N/A
Centerline overlap (mm)	8.86 (5.75–12.00)	27.28 (22.68–31.89)	< 0.001	15.59 (12.57–18.61)	< 0.001

Values are presented as mean (95% confidence interval)

HD95 = 95th percentile Hausdorff distance, HU = Hounsfield unit, N/A = not applicable due to identical values

### Diagnostic performance analysis (external validation)

To further validate the robustness of our hybrid method, quantitative evaluation was performed on the external public dataset against manually annotated reference data. The same evaluation metrics were applied for consistency.

The proposed hybrid method demonstrated good performance on the external dataset, achieving a Dice score of 0.82 (95% CI: 0.81–0.82) compared to deep learning-only (0.76, p < 0.001) and fixed HU threshold methods (0.76, p < 0.001). The method demonstrated significant improvements in vessel continuity (1.00 vs 0.73 for deep learning-only, p < 0.001) and precision (0.80 vs 0.66 for fixed HU threshold, p < 0.001). The superior performance of our hybrid method compared to deep learning-only and fixed HU threshold methods remained consistent across both datasets. Complete comparative results including all vessel structure-specific metrics are summarized in Table 3, with mean and maximum centerline distance information provided in Supplementary Table S3.

**Table 3:** Quantitative performance comparison of coronary artery segmentation methods on external validation set

Metric	Proposed hybrid method	Deep learning-only method	p value	Fixed HU threshold method	p value
Dice score	0.82 (0.81–0.82)	0.76 (0.74–0.77)	< 0.001	0.76 (0.75–0.77)	< 0.001
Recall	0.84 (0.82–0.85)	0.81 (0.79–0.83)	0.012	0.91 (0.90–0.92)	< 0.001
Precision	0.80 (0.79–0.81)	0.72 (0.69–0.74)	< 0.001	0.66 (0.64–0.68)	< 0.001
HD95 (mm)	8.68 (6.94–10.43)	19.44 (16.55–22.32)	< 0.001	8.84 (7.12–10.56)	0.476
Branch detection rate	0.88 (0.82–0.93)	1.08 (0.97–1.18)	< 0.001	0.89 (0.83–0.95)	0.210
Vessel continuity	1.00 (1.00–1.00)	0.73 (0.60–0.85)	< 0.001	1.00 (1.00–1.00)	N/A
Centerline overlap (mm)	4.66 (3.89–5.44)	16.35 (13.21–19.48)	< 0.001	4.70 (3.94–5.46)	0.461

Values are presented as mean (95% confidence interval)

HD95 = 95th percentile Hausdorff distance, HU = Hounsfield unit, N/A = not applicable due to identical values

### Subgroup analysis by stenosis degree (internal validation)

To further evaluate the robustness of our method across different pathological conditions, we conducted a subgroup analysis based on stenosis degree. Patients were categorized into two groups: < 30% stenosis and ≥ 30% stenosis. The segmentation performance showed consistent results across both groups. The Dice scores were 0.92 (95 CI: 0.91–0.93) for < 30% stenosis cases, 0.91 (95% CI: 0.89–0.93) for ≥ 30% stenosis cases. No significant differences were observed between the two groups (p = 0.27).

### Subgroup analysis by calcium score (internal validation)

An additional subgroup analysis was performed based on coronary calcium scoring to assess the impact of calcification on segmentation results. Patients were categorized into two calcium score groups: ≤ 100 and > 100. The Dice scores were 0.91 (95% CI: 0.90–0.93) for calcium score ≤ 100, 0.91 (95% CI: 0.89–0.92) for calcium score > 100. No significant differences were observed between the two groups (p = 0.72).

### Subgroup analysis by CT scanner types (internal validation)

To evaluate the robustness of our method across different imaging platforms, we conducted a subgroup analysis based on CT scanner type. Patients were categorized into two groups: SOMATOM Force and Revolution Apex. The segmentation performance showed consistent results across both scanner types. The Dice scores were 0.92 (95% CI: 0.90–0.93) for SOMATOM Force cases and 0.92 (95% CI: 0.91–0.93) for Revolution Apex cases. No significant differences were observed between the two groups (p = 0.91).

## Discussion

Our hybrid method achieved a Dice score of 0.92 on the internal validation set, significantly outperforming deep learning-only (0.68, p < 0.001) and fixed HU threshold methods (0.55, p < 0.001). External validation confirmed consistent superiority with a Dice score of 0.82 compared to deep learning-only (0.76, p < 0.001) and fixed HU threshold methods (0.76, p < 0.001).

Our design rationale addressed the specific limitations observed in existing methods through targeted solutions. First, the deep learning-only method showed lower performance primarily due to noises and vessel discontinuities, with vessel continuity metrics being furthest from the ideal value of 1. Our hybrid method achieved perfect vessel continuity scores of 1.0 (95% CI: 1.0–1.0) in both internal and external validation sets, while the deep learning-only method demonstrated poor continuity with scores of 0.07 (95% CI: − 0.12–0.25, p < 0.001) in internal validation and 0.73 (95% CI: 0.60–0.85, p < 0.001) in external validation. This poor vessel continuity led to overestimated branch detection rates of 1.22 (95% CI: 1.16–1.29) in internal validation and 1.08 (95% CI: 0.97–1.18) in external validation. Second, the fixed HU threshold method showed high dependency on scanner characteristics and imaging conditions, evidenced by significantly lower performance on the internal validation set (Dice score: 0.55, precision: 0.39, HD95: 14.83 mm, centerline overlap: 15.59 mm) compared to better results on the external validation set (Dice score: 0.76, precision: 0.66, HD95: 8.84 mm, centerline overlap: 4.7 mm). This method struggled to distinguish target lumens from surrounding tissues in distal regions due to similar HU intensities, resulting in low precision values of 0.39 (95% CI: 0.38–0.41, p < 0.001) for the internal validation set and 0.66 (95% CI: 0.64–0.68, p < 0.001) for the external validation set (Supplementary Figure S3).

Our hybrid approach addressed these limitations through the integration of deep learning with our unique mathematical integration of image processing filters. Our novel contribution was the contour mapping technique that identified inflection points more effectively than conventional edge detection methods, overcoming the limitations of fixed HU thresholds and enhancing boundary precision in complex bifurcation regions (Fig. 3). This design enabled consistent segmentation performance across diverse imaging conditions and anatomical variations, as demonstrated by our consistently superior results compared to deep learning-only and fixed HU threshold methods across both internal and external validation sets, and stable performance across different stenosis degree groups (Dice: 0.92 vs 0.91, p = 0.27), calcium score groups (Dice: 0.91 vs 0.91, p = 0.72), and CT scanner types (Dice: 0.92 vs 0.92, p = 0.91).

Beyond segmentation, the proposed method has potential clinical applications. The patient-specific 3D coronary artery segmentation output can be directly integrated into computed tomography-derived fractional flow reserve (CT-FFR) computational workflows for hemodynamic analysis. A recent clinical study has demonstrated that CT-FFR using automatic coronary segmentation can be calculated using commercially available on-site workstations with clinically acceptable diagnostic performance [36]. Our automated segmentation method could be integrated into such workflows to support routine clinical CT-FFR computation.

Despite its advantages, the method has certain limitations. Our evaluation results showed a branch detection rate of 0.81 in internal validation and 0.88 in external validation, indicating that some smaller peripheral vessels may not be fully captured in the automated segmentation (Supplementary Figure S4). Additionally, the method may fail in cases with poor CT image quality, including motion artifacts, heavy calcification, or low-dose scans. In our study, six patients were excluded due to severe motion artifacts. Supplementary Figure S5 demonstrates a representative failure case where motion artifacts during image acquisition resulted in incomplete segmentation. Future improvements could address these challenges through image quality enhancement techniques.

External validation on public datasets showed slightly lower performance compared to internal validation, primarily due to differences in ground truth annotation protocols. In some cases, ground truth segmentations extended into aortic regions (Supplementary Figure S6) and included calcified plaques (Supplementary Figure S7), while our method excluded these areas based on vessel boundary criteria.

We have not evaluated our method’s performance in complex scenarios such as calcified plaques or stented vessels, which present particular difficulties due to blooming or beam hardening artifacts [37, 38]. However, our gradient-based contour mapping approach using intensity gradients rather than fixed HU threshold may offer advantages over classical image processing methods in these challenging conditions, though future validation studies are needed to confirm this potential. Furthermore, although external validation was performed using public datasets, the relatively small sample size necessitates future validation using larger multi-center datasets to further assess the generalizability of our approach.

## Conclusions

In conclusion, we developed a novel hybrid coronary artery segmentation method integrating deep learning with our unique contour detection algorithm, demonstrating superior and consistent performance compared to deep learning-only and classical fixed HU threshold methods across diverse datasets. While limitations include incomplete detection of smaller vessels and the need for validation in complex scenarios, our method showed potential to aid CAD assessment in clinical practice.

## Supplementary Information

Below is the link to the electronic supplementary material.

Supplementary file1 (DOCX 6170 KB)

## Data Availability

The data set analyzed during the current study are not publicly available due to medical confidentiality but are available from the corresponding author on reasonable request summarized form pending the approval of the IRB.

## Abbreviations

2D: 2-Dimensional
3D: 3-Dimensional
CAD: Coronary artery disease
CCTA: Coronary computed tomography angiography
CI: Confidence interval
CT-FFR: Computed tomography-derived fractional flow reserve
HD95: 95th percentile Hausdorff Distance
HU: Hounsfield unit
IRB: Institutional Review Board
LAD: Left anterior descending artery
LCA: Left coronary artery
LCX: Left circumflex artery
RCA: Right coronary artery

## References

[1] Howson JMM, Zhao W, Barnes DR, Ho WK, Young R, Paul DS, Waite LL, Freitag DF, Fauman EB, Salfati EL, Sun BB, Eicher JD, Johnson AD, Sheu WHH, Nielsen SF, Lin WY, Surendran P, Malarstig A, Wilk JB, Tybjaerg-Hansen A, Rasmussen KL, Kamstrup PR, Deloukas P, Erdmann J, Kathiresan S, Samani NJ, Schunkert H, Watkins H, CardioGramplusC4D, Do R, Rader DJ, Johnson JA, Hazen SL, Quyyumi AA, Spertus JA, Pepine CJ, Franceschini N, Justice A, Reiner AP, Buyske S, Hindorff LA, Carty CL, North KE, Kooperberg C, Boerwinkle E, Young K, Graff M, Peters U, Absher D, Hsiung CA, Lee WJ, Taylor KD, Chen YH, Lee IT, Guo X, Chung RH, Hung YJ, Rotter JI, Juang JJ, Quertermous T, Wang TD, Rasheed A, Frossard P, Alam DS, Majumder AAS, Di Angelantonio E, Chowdhury R, Epic CVD, Chen YI, Nordestgaard BG, Assimes TL, Danesh J, Butterworth AS, Saleheen D (2017) Fifteen new risk loci for coronary artery disease highlight arterial-wall-specific mechanisms. Nat Genet 49:1113–1119 28530674 10.1038/ng.3874 PMC5555387

[2] Faccini J, Ruidavets JB, Cordelier P, Martins F, Maoret JJ, Bongard V, Ferrieres J, Roncalli J, Elbaz M, Vindis C (2017) Circulating miR-155, miR-145 and let-7c as diagnostic biomarkers of the coronary artery disease. Sci Rep 7:42916 28205634 10.1038/srep42916 PMC5311865

[3] Chong B, Jayabaskaran J, Jauhari SM, Chan SP, Goh R, Kueh MTW, Li H, Chin YH, Kong G, Anand VV, Wang JW, Muthiah M, Jain V, Mehta A, Lim SL, Foo R, Figtree GA, Nicholls SJ, Mamas MA, Januzzi JL, Chew NWS, Richards AM, Chan MY (2025) Global burden of cardiovascular diseases: projections from 2025 to 2050. Eur J Prev Cardiol 32:1001–1015 39270739 10.1093/eurjpc/zwae281

[4] Rubio PM, Garcia-Garcia HM, Sanchez AA, Alfaddagh A, Andreini D, Baggiano A, Canan A, Chen M, Chow BJW, Chelliah A, Conte E, De Cecco CN, Fairbairn T, Ferencik M, Feuchtner G, Foellmer B, Fuss C, Gransar H, Hamdan A, Han BK, Jamieson S, Ko B, Leipsic JA, Madan N, Michallek F, Mushtaq S, Nagpal P, Ng MY, Nicol E, Niinuma H, Pontone G, Pourmorteza A, Reid A, Weir-McCall JR, Whelton S, Williams M, Yu J, Arbab-Zadeh A (2025) Key advances in cardiac computed tomography: a review of the most relevant studies published in 2024 on coronary and structural heart disease. J Cardiovasc Comput Tomogr 19:291–298 40300917 10.1016/j.jcct.2025.04.008

[5] Nannini G, Saitta S, Baggiano A, Maragna R, Mushtaq S, Pontone G, Redaelli A (2024) A fully automated deep learning approach for coronary artery segmentation and comprehensive characterization. APL Bioeng 8:016103 38269204 10.1063/5.0181281 PMC10807932

[6] Park D, Park EA, Jeong B, Lee W (2024) A comparative analysis of deep learning-based location-adaptive threshold method software against other commercially available software. Int J Cardiovasc Imaging 40:1269–1281 38634943 10.1007/s10554-024-03099-7 PMC11213768

[7] Moeskops P, Wolterink JM, van der Velden BH, Gilhuijs KG, Leiner T, Viergever MA, Išgum I (2016) Deep learning for multi-task medical image segmentation in multiple modalities. In: International Conference on Medical Image Computing and Computer-Assisted Intervention. Springer, pp 478–486

[8] Singh G, Al’Aref SJ, Van Assen M, Kim TS, van Rosendael A, Kolli KK, Dwivedi A, Maliakal G, Pandey M, Wang J, Do V, Gummalla M, De Cecco CN, Min JK (2018) Machine learning in cardiac CT: basic concepts and contemporary data. J Cardiovasc Comput Tomogr 12:192–201 29754806 10.1016/j.jcct.2018.04.010

[9] Tian Y, Pan Y, Duan F, Zhao S, Wang Q, Wang W (2016) Automated segmentation of coronary arteries based on statistical region growing and heuristic decision method. Biomed Res Int 2016:3530251 27872849 10.1155/2016/3530251 PMC5107877

[10] Muscogiuri G, Van Assen M, Tesche C, De Cecco CN, Chiesa M, Scafuri S, Guglielmo M, Baggiano A, Fusini L, Guaricci AI, Rabbat MG, Pontone G (2020) Artificial intelligence in coronary computed tomography angiography: from anatomy to prognosis. Biomed Res Int 2020:6649410 33381570 10.1155/2020/6649410 PMC7762640

[11] Molenaar MA, Selder JL, Nicolas J, Claessen BE, Mehran R, Bescos JO, Schuuring MJ, Bouma BJ, Verouden NJ, Chamuleau SAJ (2022) Current state and future perspectives of artificial intelligence for automated coronary angiography imaging analysis in patients with ischemic heart disease. Curr Cardiol Rep 24:365–376 35347566 10.1007/s11886-022-01655-y PMC8979928

[12] Wang Q, Xu L, Wang L, Yang X, Sun Y, Yang B, Greenwald SE (2023) Automatic coronary artery segmentation of CCTA images using UNet with a local contextual transformer. Front Physiol 14:1138257 37675283 10.3389/fphys.2023.1138257 PMC10478234

[13] Wang J, Chen Q, Jiang X, Zhang Z, Tang Z (2025) Segmentation of coronary artery and calcification using prior knowledge based deep learning framework. Med Phys 52:3030–3043 39878608 10.1002/mp.17642 PMC12082760

[14] Shin CI, Park SJ, Kim JH, Yoon YE, Park EA, Koo BK, Lee W (2021) Coronary artery lumen segmentation using location-adaptive threshold in coronary computed tomographic angiography: a proof-of-concept. Korean J Radiol 22:688–696 33543843 10.3348/kjr.2020.0296 PMC8076829

[15] Gebhard C, Fuchs TA, Fiechter M, Stehli J, Stahli BE, Gaemperli O, Kaufmann PA (2013) Image quality of low-dose CCTA in obese patients: impact of high-definition computed tomography and adaptive statistical iterative reconstruction. Int J Cardiovasc Imaging 29:1565–1574 23624958 10.1007/s10554-013-0228-4

[16] Komatsu S, Kamata T, Imai A, Ohara T, Takewa M, Ohe R, Miyaji K, Yoshida J, Kodama K (2013) Coronary computed tomography angiography using ultra-low-dose contrast media: radiation dose and image quality. Int J Cardiovasc Imaging 29:1335–1340 23440348 10.1007/s10554-013-0201-2

[17] Wang C, Frimmel H, Persson A, Smedby Ö (2008) An interactive software module for visualizing coronary arteries in CT angiography. Int J Comput Assist Radiol Surg 3:11–18

[18] Kitamura Y, Li Y, Ito W, Ishikawa H (2014) Coronary lumen and plaque segmentation from CTA using higher-order shape prior. Med Image Comput Comput Assist Interv 17:339–347 25333136 10.1007/978-3-319-10404-1_43

[19] Ghanem AM, Hamimi AH, Matta JR, Carass A, Elgarf RM, Gharib AM, Abd-Elmoniem KZ (2019) Automatic coronary wall and atherosclerotic plaque segmentation from 3D coronary CT angiography. Sci Rep 9:47 30631101 10.1038/s41598-018-37168-4 PMC6328572

[20] Li Y, Wu Y, He J, Jiang W, Wang J, Peng Y, Jia Y, Xiong T, Jia K, Yi Z, Chen M (2022) Automatic coronary artery segmentation and diagnosis of stenosis by deep learning based on computed tomographic coronary angiography. Eur Radiol 32:6037–6045 35394183 10.1007/s00330-022-08761-z

[21] Gharleghi R, Adikari D, Ellenberger K, Ooi SY, Ellis C, Chen CM, Gao R, He Y, Hussain R, Lee CY, Li J, Ma J, Nie Z, Oliveira B, Qi Y, Skandarani Y, Vilaca JL, Wang X, Yang S, Sowmya A, Beier S (2022) Automated segmentation of normal and diseased coronary arteries—the ASOCA challenge. Comput Med Imaging Graph 97:102049 35334316 10.1016/j.compmedimag.2022.102049

[22] Milletari F, Navab N, Ahmadi S-A (2016) V-net: fully convolutional neural networks for volumetric medical image segmentation. In: 2016 fourth international conference on 3D vision (3DV). IEEE, pp 565–571

[23] Zhang X, Du H, Song G, Bao F, Zhang Y, Wu W, Liu P (2022) X-ray coronary centerline extraction based on C-UNet and a multifactor reconnection algorithm. Comput Methods Programs Biomed 226:107114 36116399 10.1016/j.cmpb.2022.107114

[24] Lee JO, Park EA, Park D, Lee W (2023) Deep learning-based automated quantification of coronary artery calcification for contrast-enhanced coronary computed tomographic angiography. J Cardiovasc Dev Dis. 10.3390/jcdd10040143 37103022 10.3390/jcdd10040143 PMC10146297

[25] Antiga L, Ene-Iordache B, Remuzzi A (2003) Computational geometry for patient-specific reconstruction and meshing of blood vessels from MR and CT angiography. IEEE Trans Med Imaging 22:674–684 12846436 10.1109/TMI.2003.812261

[26] Ito K (2000) Gaussian filter for nonlinear filtering problems. In: Proceedings of the 39th IEEE Conference on Decision and Control (Cat No 00CH37187). IEEE, pp 1218–1223

[27] He K, Sun J, Tang X (2013) Guided image filtering. IEEE Trans Pattern Anal Mach Intell 35:1397–1409 23599054 10.1109/TPAMI.2012.213

[28] Mitchell DP (1987) Generating antialiased images at low sampling densities. ACM SIGGRAPH Computer Graphics 21:65–72

[29] Ziou D, Tabbone S (1998) Edge detection techniques-an overview. Pattern Recognition and Image Analysis C/C of Raspoznavaniye Obrazov I Analiz Izobrazhenii 8:537–559

[30] Chudasama D, Patel T, Joshi S, Prajapati GI (2015) Image segmentation using morphological operations. Int J Comput Appl. 10.5120/20654-3197

[31] Khudeev R (2005) A new flood-fill algorithm for closed contour. In: 2005 Siberian conference on control and communications. IEEE, pp 172–176

[32] Mintz GS, Potkin BN, Keren G, Satler LF, Pichard AD, Kent KM, Popma JJ, Leon MB (1992) Intravascular ultrasound evaluation of the effect of rotational atherectomy in obstructive atherosclerotic coronary artery disease. Circulation 86:1383–1393 1423950 10.1161/01.cir.86.5.1383

[33] Haralick RM, Sternberg SR, Zhuang X (1987) Image analysis using mathematical morphology. IEEE Trans Pattern Anal Mach Intell. 10.1109/tpami.1987.4767941 21869411 10.1109/tpami.1987.4767941

[34] Rodríguez JE, Ayala D (2001) Erosion and dilation on 2-D and 3-D digital images: a new size-independent approach. In: VMV, p 143

[35] Nasr-Esfahani E, Karimi N, Jafari MH, Soroushmehr SMR, Samavi S, Nallamothu B, Najarian K (2018) Segmentation of vessels in angiograms using convolutional neural networks. Biomed Signal Process Control 40:240–251

[36] Hwang D, Park SH, Nam CW, Doh JH, Kim HK, Kim Y, Chun EJ, Koo BK (2024) Diagnostic performance of on-site automatic coronary computed tomography angiography-derived fractional flow reserve. Korean Circ J 54:382–394 38767442 10.4070/kcj.2023.0288 PMC11252635

[37] Lin M, Lan Q, Huang C, Yang B, Yu Y (2024) Wavelet-based U-shape network for bioabsorbable vascular stents segmentation in IVOCT images. Front Physiol 15:1454835 39210969 10.3389/fphys.2024.1454835 PMC11358552

[38] Zhao H, Zhu W, Jin L, Xiong Y, Deng X, Li Y, Zou W (2025) Calcium deblooming in coronary computed tomography angiography via semantic-oriented generative adversarial network. Comput Med Imaging Graph 122:102515 40020506 10.1016/j.compmedimag.2025.102515

