# Supplementary material for: A novel hybrid segmentation method coupled with deep learning for coronary artery extraction from coronary CT angiography
Source: Int J Cardiovasc Imaging. 2026 Feb 26;42(5):965–76. doi: 10.1007/s10554-026-03643-7 (PMC13136194; doi:10.1007/s10554-026-03643-7)
Supplement: Supplementary file 1 — Supplementary file1 (DOCX 6170 KB) [file 10554_2026_3643_MOESM1_ESM.docx]

**Supplementary Material**

**A Novel Hybrid Segmentation Method Coupled with Deep Learning for Coronary Artery Extraction from Coronary CT Angiography**

**Contents**

**I. Supplementary Methods**

Supplementary Method S1. V-Net training dataset details.

Supplementary Method S2. Component connection and noise removal details.

Supplementary Method S3. Adaptive window generation for coronary artery segmentation.

Supplementary Method S4. Morphological smoothing for 3-dimensional coronary artery segmentation output.

**II. Supplementary Equations**

Supplementary Equation S1. Gaussian and weighted average filters.

Supplementary Equation S2. Laplacian filter.

Supplementary Equation S3. Upper and lower bounds.

Supplementary Equation S4. Dice score.

**III. Supplementary Tables**

Supplementary Table S1. Patient-level data showing stenosis severity, calcium scores, and CT scanner specifications.

Supplementary Table S2. Coronary computed tomography angiography image acquisition parameters and specifications.

Supplementary Table S3. Centerline distance information of different segmentation methods.

**IV. Supplementary Figures**

Supplementary Figure S1. Generation of cross-sectional planes using the centerline and an adaptive window.

Supplementary Figure S2. Direct comparison of original scan, ground-truth segmentation, and proposed method results.

Supplementary Figure S3. Segmentation errors of fixed Hounsfield unit threshold method in distal coronary regions.

Supplementary Figure S4. Representative failure case demonstrating incomplete detection of smaller peripheral vessels.

Supplementary Figure S5. Representative case demonstrating patient exclusion due to severe motion artifacts.

Supplementary Figure S6. Segmentation results of ground truth and our hybrid method in aortic regions.

Supplementary Figure S7. Segmentation results of ground truth and our hybrid method in calcified regions.

**I. Supplementary Methods**

**Supplementary Method S1. V-Net training dataset details.**

The V-Net training dataset comprised 231,219 manually segmented coronary artery CT images from 457 patients (mean age 62.3±9.4 years, 74.8% male) collected from five institutions in South Korea: Seoul National University Hospital, Ulsan University Hospital, Keimyung University Hospital, Inje University Hospital, and Pusan National University Hospital.

Manual segmentation was performed by medical imaging experts with more than 5 years of experience. The dataset was split at the patient level into training (n=365, 80%), validation (n=46, 10%), and internal testing (n=46, 10%) sets.

**Supplementary Method S2. Component connection and noise removal details.**

For each disconnected component, we constructed node sets representing the main centerline and interrupted segments, then calculated 3-dimensional (3D) Euclidean distances between nodes to identify potential connection points. However, if the distance between two nodes is large, reconnection is not considered [1]. Components were connected when the intensity along the connection path remained within 0.5 [2] to 1.45 times [3] the component mean intensity, preventing erroneous connections through background or calcium deposits. We calculated the bending angle of potential connection paths and rejected reconnections when the angle was less than 135°, as this would be inconsistent with realistic vessel anatomy [1]. Next, venous structures were identified and removed based on anatomical trajectory patterns, where vessels running retrograde from the left anterior descending artery (LAD) or crossing the left circumflex artery (LCX) horizontally were classified as veins. Finally, two largest components identified as left coronary artery (LCA) and right coronary artery (RCA) were obtained, and remaining small disconnected components were subsequently removed as noise.

**Supplementary Method S3. Adaptive window generation for coronary artery segmentation.**

To facilitate coronary artery segmentation and prevent segmentation from extending to surrounding non-target structures, an adaptive window was applied within each cross-sectional plane to eliminate non-target regions. This process employed a sequence of digital image processing filters. Initially, Gaussian and weighted average filters were applied to the original image to reduce noise (Supplementary Equation S1) [4-6]. Subsequently, a Laplacian filter, based on the second derivative, was applied to enhance the edge definition of the target lumen, as the pixel gradient was locally maximized along the contour (Supplementary Equation S2) [5, 7]. The sequential application of filters follows established theoretical principles: noise reduction must precede edge enhancement to ensure accurate vessel boundary detection, as applying edge enhancement without prior noise reduction would result in unwanted enhancement of noise artifacts within the vessel lumen, compromising boundary detection accuracy. This sequential application of filters isolated the target lumen from the background (Supplementary Figure S1d). The adaptive window was then defined using the maximum and minimum coordinates of the isolated lumen in the 2-dimensional (2D) XY plane (Supplementary Figure S1e).

**Supplementary Method S4. Morphological smoothing for 3-dimensional coronary artery segmentation output.**

The reshaped lumens from all cross-sectional planes were stacked to reconstruct the 3D coronary artery segmentation output. After segmentation, morphological smoothing techniques were applied to eliminate surface irregularities and fill potential holes in the 3D coronary artery segmentation output, as cross-sectional processing resulted in surface discontinuities. The smoothing process combines erosion and dilation algorithms [8-10], which are widely utilized in digital image processing. The final segmented 3D coronary artery segmentation output is shown in Figure 5.

**II. Supplementary Equations**

**Supplementary Equation S1. Gaussian and weighted average filters.**

Gaussian and weighted average filters were applied to the original image to reduce noise, following the equations:

,

$g(x,y)=\frac{\sum_{s=-a}^{a} \sum_{t=-b}^{b} w(s,t)f(x+s,y+t)}{\sum_{s=-a}^{a} \sum_{t=-b}^{b} w(s,t)}$,

where σ, w, and f represent the standard deviation of the Gaussian function, weight coefficients, and pixel values of the image, respectively.

**Supplementary Equation S2. Laplacian filter.**

A Laplacian filter, based on the second derivative, was applied to enhance the edge definition of the target lumen, as the pixel gradient was locally maximized along the contour:

$\nabla^{2}f=\frac{\partial^{2}f}{\partial x^{2}}+\frac{\partial^{2}f}{\partial y^{2}}$ .

**Supplementary Equation S3. Upper and lower bounds.**

For each intensity group (low-intensity: < 0.5 times seed intensity; high-intensity: > 1.45 times seed intensity), the upper and lower bounds were calculated using the standard deviation of the respective contour pixel values:

$Bounds=\frac{\sum_{i=1}^{n} X_{i}}{n}\pm k\sqrt{\frac{\sum_{i=1}^{n} (X_{i}-\bar{X})^{2}}{n}}$,

where k was a constant, and X and n represented the original values and the number of detected contour pixels within each intensity group, respectively.

**Supplementary Equation S4. Dice score.**

The Dice score quantifies the overall overlap between predicted and ground truth segmentations and was computed as follows:

$Dice=\frac{2TP}{2TP+FP+FN}$,

where TP, FN, and FP represent true positives, false negatives, and false positives, respectively.

**III. Supplementary Tables**

**Supplementary Table S1. Patient-level data showing stenosis severity, calcium scores, and CT scanner specifications.**

| Case | Stenosis severity | Stenosis degree | Calcium score | Calcium group | CT scanner |
| --- | --- | --- | --- | --- | --- |
| 1 | Normal | <30% | 0 | ≤100 | Revolution Apex |
| 2 | Normal | <30% | 0 | ≤100 | Revolution Apex |
| 3 | Normal | <30% | 0 | ≤100 | Revolution Apex |
| 4 | Normal | <30% | 0 | ≤100 | Revolution Apex |
| 5 | Normal | <30% | 0 | ≤100 | Revolution Apex |
| 6 | Normal | <30% | 0 | ≤100 | Revolution Apex |
| 7 | Normal | <30% | 0 | ≤100 | Revolution Apex |
| 8 | Normal | <30% | 0 | ≤100 | Revolution Apex |
| 9 | Normal | <30% | 0 | ≤100 | Revolution Apex |
| 10 | Normal | <30% | 0 | ≤100 | Revolution Apex |
| 11 | Normal | <30% | 0 | ≤100 | Revolution Apex |
| 12 | Normal | <30% | 0 | ≤100 | Revolution Apex |
| 13 | Normal | <30% | 0 | ≤100 | Revolution Apex |
| 14 | Normal | <30% | 0 | ≤100 | Revolution Apex |
| 15 | Normal | <30% | 0 | ≤100 | Revolution Apex |
| 16 | Mild atherosclerosis | <30% | 66 | ≤100 | Revolution Apex |
| 17 | Mild atherosclerosis | <30% | 16.5 | ≤100 | Revolution Apex |
| 18 | Mild atherosclerosis | ≥30% | 201.6 | >100 | Revolution Apex |
| 19 | Mild atherosclerosis | ≥30% | 269 | >100 | Revolution Apex |
| 20 | Mild atherosclerosis | <30% | 97.7 | ≤100 | Revolution Apex |
| 21 | Mild atherosclerosis | ≥30% | 117.7 | >100 | Revolution Apex |
| 22 | Mild atherosclerosis | ≥30% | 184.3 | >100 | Revolution Apex |
| 23 | Mild atherosclerosis | ≥30% | 116.5 | >100 | Revolution Apex |
| 24 | Mild atherosclerosis | ≥30% | 137.5 | >100 | Revolution Apex |
| 25 | Mild atherosclerosis | ≥30% | 98.2 | ≤100 | Revolution Apex |
| 26 | Mild atherosclerosis | <30% | 166 | >100 | Revolution Apex |
| 27 | Mild atherosclerosis | ≥30% | 296.1 | >100 | Revolution Apex |
| 28 | Mild atherosclerosis | <30% | 81.8 | ≤100 | Revolution Apex |
| 29 | Mild atherosclerosis | <30% | 15.8 | ≤100 | Revolution Apex |
| 30 | Severe atherosclerosis | <30% | 565.6 | >100 | Revolution Apex |
| 31 | Severe atherosclerosis | ≥30% | 732.8 | >100 | Revolution Apex |
| 32 | Severe atherosclerosis | ≥30% | 639.7 | >100 | Revolution Apex |
| 33 | Severe atherosclerosis | ≥30% | 2639.7 | >100 | Revolution Apex |
| 34 | Severe atherosclerosis | ≥30% | 699.2 | >100 | Revolution Apex |
| 35 | Severe atherosclerosis | ≥30% | 642.5 | >100 | Revolution Apex |
| 36 | Severe atherosclerosis | ≥30% | 1259.9 | >100 | Revolution Apex |
| 37 | Severe atherosclerosis | ≥30% | 282.9 | >100 | Revolution Apex |
| 38 | Severe atherosclerosis | ≥30% | 528.4 | >100 | Revolution Apex |
| 39 | Severe atherosclerosis | ≥30% | 714.3 | >100 | Revolution Apex |
| 40 | Severe atherosclerosis | ≥30% | 158.9 | >100 | Revolution Apex |
| 41 | Severe atherosclerosis | ≥30% | 96 | ≤100 | Revolution Apex |
| 42 | Severe atherosclerosis | ≥30% | 120.3 | >100 | Revolution Apex |
| 43 | Severe atherosclerosis | ≥30% | 193.4 | >100 | Revolution Apex |
| 44 | Severe atherosclerosis | ≥30% | 65.4 | ≤100 | Revolution Apex |
| 45 | Normal | <30% | 0 | ≤100 | Revolution Apex |
| 46 | Normal | <30% | 0 | ≤100 | SOMATOM Force |
| 47 | Normal | <30% | 0 | ≤100 | SOMATOM Force |
| 48 | Normal | <30% | 0 | ≤100 | SOMATOM Force |
| 49 | Normal | <30% | 0 | ≤100 | SOMATOM Force |
| 50 | Normal | <30% | 0 | ≤100 | SOMATOM Force |
| 51 | Normal | <30% | 0 | ≤100 | SOMATOM Force |
| 52 | Normal | <30% | 0 | ≤100 | SOMATOM Force |
| 53 | Normal | <30% | 0 | ≤100 | SOMATOM Force |
| 54 | Normal | <30% | 0 | ≤100 | SOMATOM Force |
| 55 | Normal | <30% | 0 | ≤100 | SOMATOM Force |
| 56 | Normal | <30% | 0 | ≤100 | SOMATOM Force |
| 57 | Normal | <30% | 0 | ≤100 | SOMATOM Force |
| 58 | Normal | <30% | 0 | ≤100 | SOMATOM Force |
| 59 | Mild atherosclerosis | ≥30% | 298.9 | >100 | SOMATOM Force |
| 60 | Mild atherosclerosis | ≥30% | 296.6 | >100 | SOMATOM Force |
| 61 | Mild atherosclerosis | ≥30% | 171.7 | >100 | SOMATOM Force |
| 62 | Mild atherosclerosis | ≥30% | 46.7 | ≤100 | SOMATOM Force |
| 63 | Mild atherosclerosis | <30% | 18.2 | ≤100 | SOMATOM Force |
| 64 | Mild atherosclerosis | <30% | 253.7 | >100 | SOMATOM Force |
| 65 | Mild atherosclerosis | <30% | 39 | ≤100 | SOMATOM Force |
| 66 | Mild atherosclerosis | <30% | 186.3 | >100 | SOMATOM Force |
| 67 | Mild atherosclerosis | <30% | 255.8 | >100 | SOMATOM Force |
| 68 | Mild atherosclerosis | <30% | 63.3 | ≤100 | SOMATOM Force |
| 69 | Mild atherosclerosis | <30% | 50 | ≤100 | SOMATOM Force |
| 70 | Mild atherosclerosis | <30% | 270 | >100 | SOMATOM Force |
| 71 | Mild atherosclerosis | <30% | 270.9 | >100 | SOMATOM Force |
| 72 | Severe atherosclerosis | <30% | 348.5 | >100 | SOMATOM Force |
| 73 | Severe atherosclerosis | <30% | 88.9 | ≤100 | SOMATOM Force |
| 74 | Severe atherosclerosis | ≥30% | 105.6 | >100 | SOMATOM Force |
| 75 | Severe atherosclerosis | ≥30% | 965.2 | >100 | SOMATOM Force |
| 76 | Severe atherosclerosis | ≥30% | 379.5 | >100 | SOMATOM Force |
| 77 | Severe atherosclerosis | ≥30% | 1507.3 | >100 | SOMATOM Force |
| 78 | Severe atherosclerosis | ≥30% | 1362.8 | >100 | SOMATOM Force |
| 79 | Severe atherosclerosis | ≥30% | 95.9 | >100 | SOMATOM Force |
| 80 | Severe atherosclerosis | ≥30% | 306.4 | >100 | SOMATOM Force |
| 81 | Severe atherosclerosis | ≥30% | 882.8 | >100 | SOMATOM Force |
| 82 | Severe atherosclerosis | ≥30% | 1017.7 | >100 | SOMATOM Force |
| 83 | Severe atherosclerosis | ≥30% | 886.2 | >100 | SOMATOM Force |
| 84 | Severe atherosclerosis | ≥30% | 690 | >100 | SOMATOM Force |

**Supplementary Table S2. Coronary computed tomography angiography image acquisition parameters and specifications.**

| Parameter | SOMATOM Force | Revolution Apex |
| --- | --- | --- |
| Scanner model | SOMATOM Force | Revolution Apex |
| Tube voltage range | 70-120 kVp | 70-120 kVp |
| Slice thickness | 0.75 mm | 0.625 mm |
| In-plane resolution | 0.31-0.39 mm | 0.23 mm |
| Voxel size range | 0.07-0.11 mm | 0.1-0.5 mm |
| Reconstruction kernel | Bv40 ADMIRE 3 | Standard kernel |
| Contrast protocol | Contrast 50cc/4.0ml/cc / Contrast + Saline 6cc+32cc 4.0ml/cc | Contrast 45cc/4.5ml/cc / Contrast + Saline 6cc+32cc 4.0ml/cc |
| Intensity range/clipping | -1024~3071HU, clipping | -1024~3071HU, clipping |
| Typical image matrix | 512×512×(219-343) | 512×512×(192-256) |

kVp = kilovoltage peak**Supplementary Table S3. Centerline distance information of different segmentation methods.**

|  | Internal validation set | | External validation set | |
| --- | --- | --- | --- | --- |
| Method | Mean (mm) | Maximum (mm) | Mean (mm) | Maximum (mm) |
| Groud truth | 732.76  (705.15-760.38) | 1038.60 | 621.26  (582.31-660.20) | 829.86 |
| Proposed hybrid method | 659.34  (633.83-684.85) | 977.21 | 537.89  (506.04-569.74) | 767.31 |
| Deep learning-only method | 805.57  (771.30-839.84) | 1207.60 | 600.06  (568.62-631.50) | 841.99 |
| Fixed HU threshold method | 631.83  (604.49-659.16) | 942.03 | 538.24  (506.12-570.36) | 768.26 |

Values are presented as mean (95% confidence interval). HU = Hounsfield unit

**IV. Supplementary Figures**

**Supplementary Figure S1. Generation of cross-sectional planes using the centerline and an adaptive window.** (a) A representative example of extracting a cross sectional plane from the centerline. (b) Cross-sectional plane extracted from the centerline. (c) The cross-sectional plane with the target lumen at the center, represented by a yellow dotted rectangle. (d) The result of sequentially applying Gaussian, weighted average, and Laplacian filters. White and black indicate positive and negative pixel values, respectively. (e) An extracted adaptive window based on the coordinates of the isolated positive area in (d), which contains the seed point. The red dot represents the seed point derived from the centerline information.

**Supplementary Figure S2. Direct comparison of original scan, ground-truth segmentation, and proposed method results.**

(a) Original axial coronary computed tomography angiography scan showing coronary vessels. (b) Ground-truth manual segmentation. (c) Proposed hybrid method segmentation result.

**Supplementary Figure S3. Segmentation errors of fixed Hounsfield unit threshold method in distal coronary regions.**

**Supplementary Figure S4. Representative failure case demonstrating incomplete detection of smaller peripheral vessels.**

(a) Automated 3-dimensional segmentation output showing missing small branch (red circle). (b) Ground truth segmentation demonstrating the presence of the small peripheral vessel. (c-e) Detailed views of the missed small branch in axial (c), coronal (d), and sagittal (e) views. Red circles highlight the small peripheral vessel that was not captured by the automated segmentation but is present in the manual reference annotation.

**Supplementary Figure S5. Representative case demonstrating patient exclusion due to severe motion artifacts.**

(a) 3-dimensional reconstruction showing fragmented vessel appearance due to motion artifacts (red circle). (b-d) Motion artifacts demonstrated in axial (b), coronal (c), and sagittal (d) views. Red circles highlight areas where motion artifacts during image acquisition resulted in vessel discontinuity and image degradation.

**Supplementary Figure S6. Segmentation results of ground truth and our hybrid method in aortic regions.**

(a) Original CT image showing aortic region. (b) Ground truth segmentation. (c) Hybrid method segmentation.

**Supplementary Figure S7. Segmentation results of ground truth and our hybrid method in calcified regions.**

(a) Original CT image showing calcified coronary artery. (b) Ground truth segmentation. (c) Hybrid method segmentation.

**Supplementary References**

1. Zhang X, Du H, Song G, Bao F, Zhang Y, Wu W, Liu P (2022) X-ray coronary centerline extraction based on C-UNet and a multifactor reconnection algorithm. Comput Methods Programs Biomed 226:107114.

2. Park D, Park EA, Jeong B, Lee W (2024) A comparative analysis of deep learning-based location-adaptive threshold method software against other commercially available software. Int J Cardiovasc Imaging 40:1269-1281.

3. Lee JO, Park EA, Park D, Lee W (2023) Deep Learning-Based Automated Quantification of Coronary Artery Calcification for Contrast-Enhanced Coronary Computed Tomographic Angiography. J Cardiovasc Dev Dis 10.

4. Ito K (2000) Gaussian filter for nonlinear filtering problems. Proceedings of the 39th IEEE Conference on Decision and Control (Cat No 00CH37187). IEEE, pp 1218-1223.

5. He K, Sun J, Tang X (2013) Guided image filtering. IEEE Trans Pattern Anal Mach Intell 35:1397-1409.

6. Mitchell DP (1987) Generating antialiased images at low sampling densities. ACM SIGGRAPH Computer Graphics 21:65-72.

7. Ziou D, Tabbone S (1998) Edge detection techniques-an overview. Pattern Recognition and Image Analysis C/C of Raspoznavaniye Obrazov I Analiz Izobrazhenii 8:537-559.

8. Chudasama D, Patel T, Joshi S, Prajapati GI (2015) Image segmentation using morphological operations. International Journal of Computer Applications 117.

9. Haralick RM, Sternberg SR, Zhuang X (1987) Image analysis using mathematical morphology. IEEE transactions on pattern analysis and machine intelligence:532-550.

10. Rodríguez JE, Ayala D (2001) Erosion and Dilation on 2-D and 3-D Digital Images: A New Size-Independent Approach. VMV, p 143.
